# Supplementary material for: Tumor-derived NKG2D ligand sMIC reprograms NK cells to an inflammatory phenotype through CBM signalosome activation
Source: Commun Biol. 2021 Jul 22;4:905. doi: 10.1038/s42003-021-02440-3 (PMC8298432; doi:10.1038/s42003-021-02440-3)
Supplement: Supplementary file 2 — Supplementary Information [file 42003_2021_2440_MOESM2_ESM.pdf]

Supplementary Fig. 1

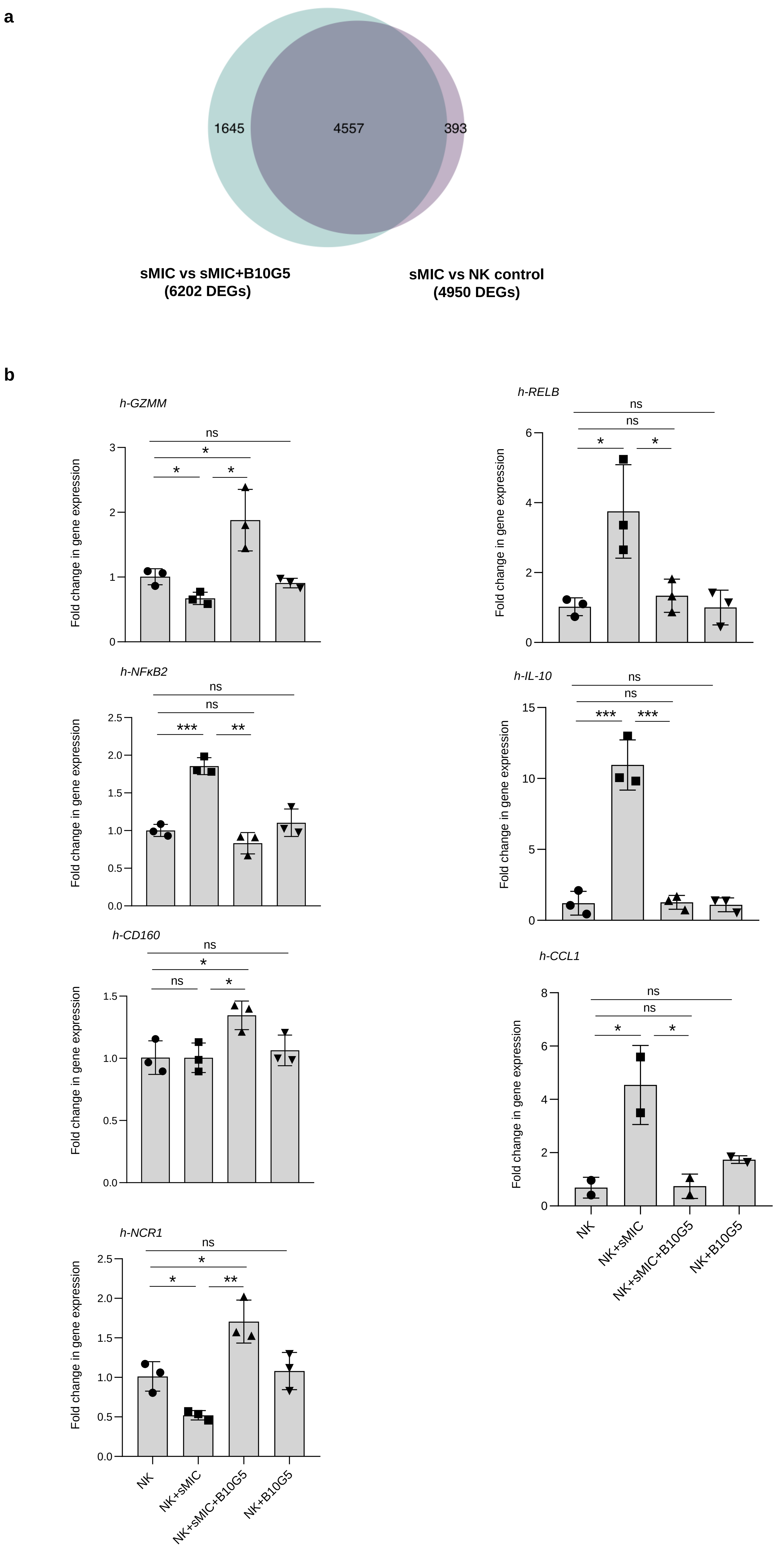

Supplementary Fig. 1: (a) Venn diagram demonstrating the genes differentially regulated by sMIC and sMIC+B10G5 and the overlapping genes between the groups in human NK cells. **(b)** Validation of changes in the expression of genes in human NK cells identified by bulk RNA sequencing (Fig 1c) by qRT-PCR. * represents $p < 0.05$,** represents $p < 0.01$, *** represents $p < 0.001$(Student's t-test; two tailed)

Supplementary Fig. 2

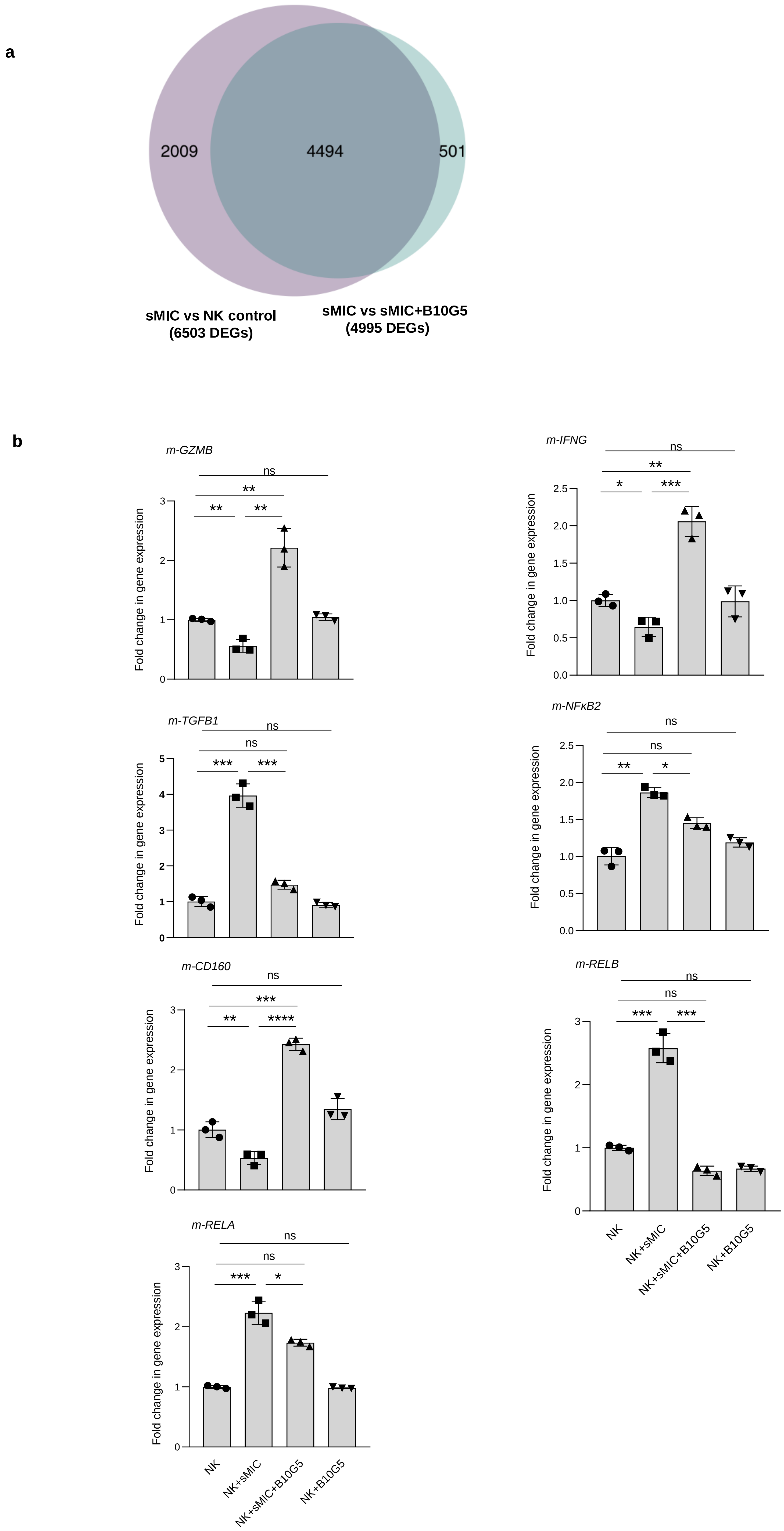

Supplementary Fig. 2: (a) Venn diagram demonstrating the genes differentially regulated by sMIC and sMIC+B10G5 and the overlapping genes between the groups in mouse NK cells. **(b)** Validation of changes in the expression of genes in mouse NK cells identified by bulk RNA sequencing (Fig. 1d) by qRT-PCR. * represents p< 0.05,** represents p<0.01, *** represents p< 0.001 and **** represents p< 0.0001 (Student's t-test; two tailed)

Supplementary Fig. 3

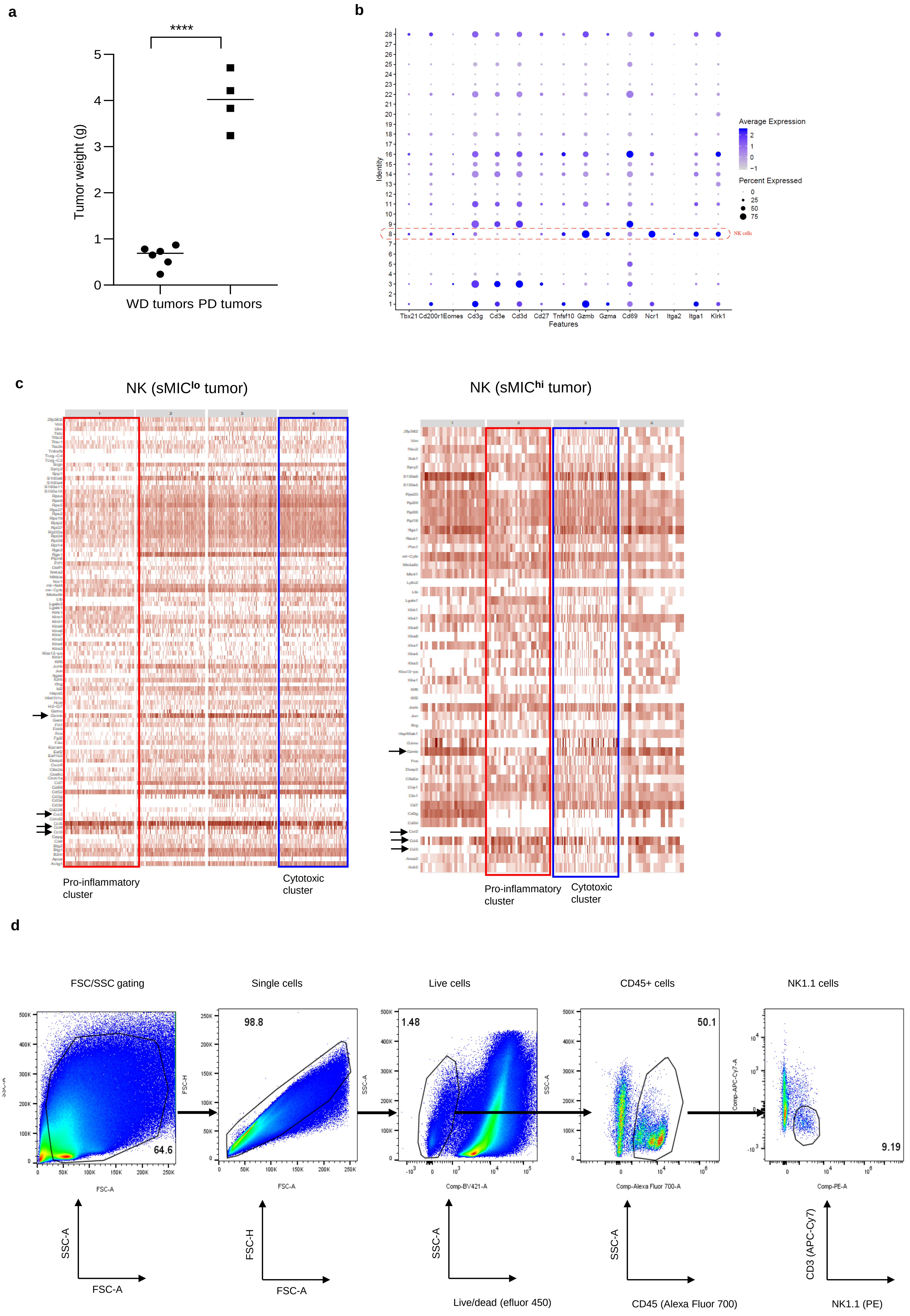

Supplementary Fig. 3: Single cell expression profiles of tumor –infiltrating NK cells reveal distinct functional phenotypes associated with tumor progression in TRAMP/MICB mice.
(a) Comparison of weights of well differentiated sMIC^{lo} and poorly differentiated sMIC^{hi} tumors from TRAMP/MICB mice at necropsy. **** p<0.0001 (Student's t-test; two tailed) **(b)** Annotation of NK cell population based on prototypic cell surface and functional markers. **(c)** Heatmaps illustrating differentially expressed genes and the annotated cytotoxic and pro-inflammatory clusters in NK cells in sMIC^{lo} and sMIC^{hi} tumors using HIPPO method. **(d)** Representative flow cytometry gating strategy for identifying tumor infiltrating NK cells for **Fig 2h.**

Supplementary Fig. 4

a.

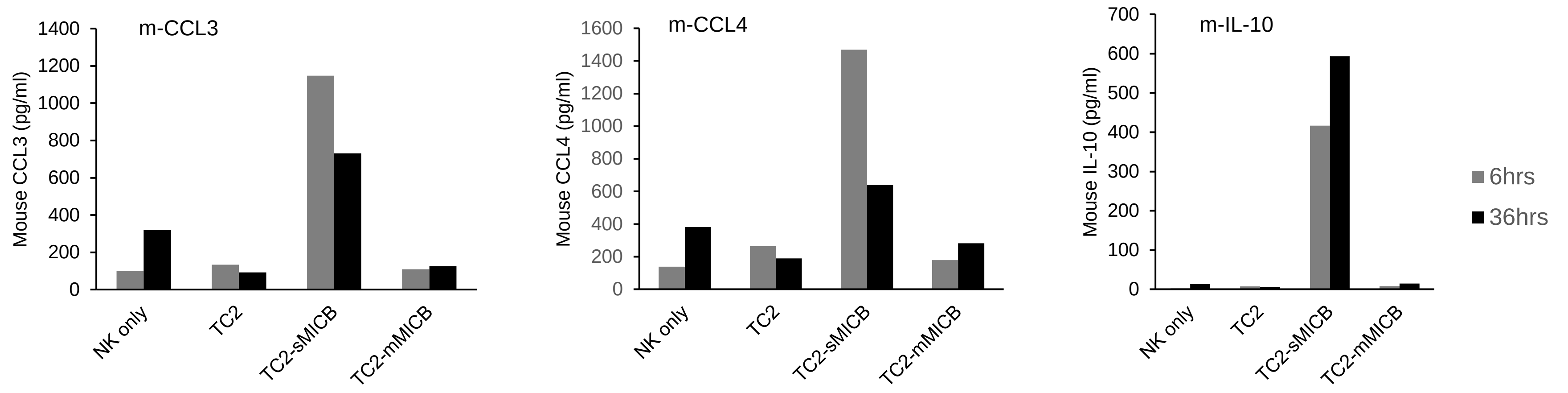

b.

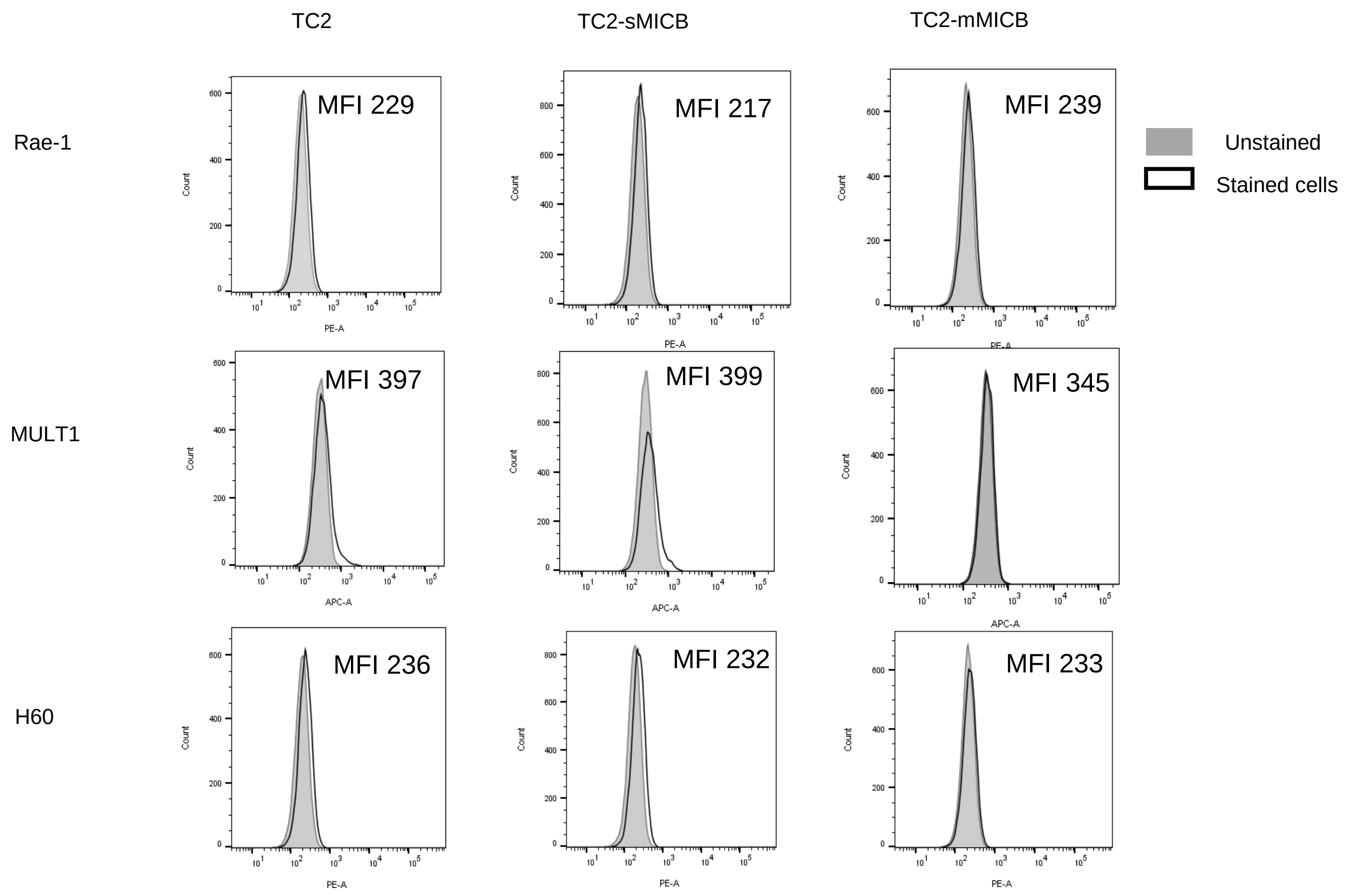

d.

e.

f.

g.

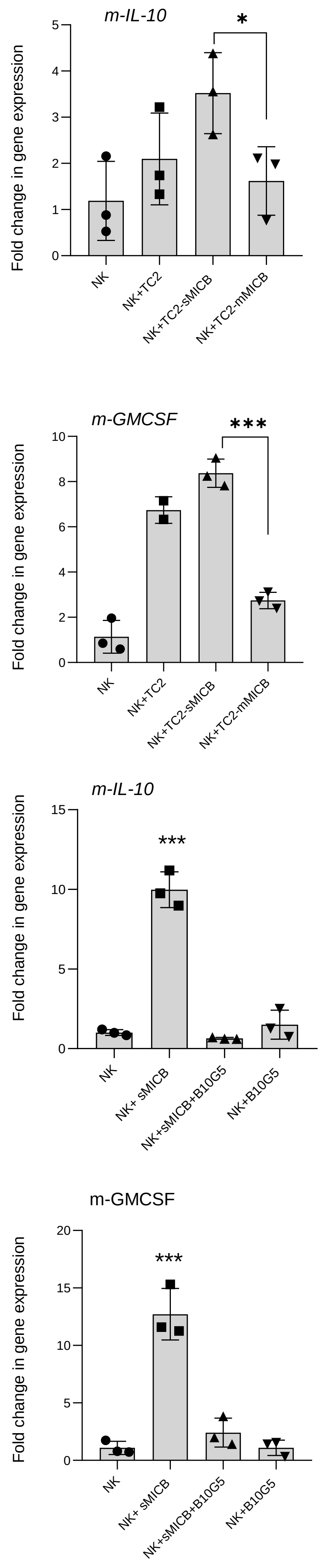

c.

Cytokine/Chemokine (pg/ml)	TC2	TC2-sMICB	TC2-mMICB
**IL-10**	0.64	OOR<	OOR<
**GMCSF**	6.21	OOR<	OOR<
**CCL4**	OOR<	OOR<	OOR<
**CCL3**	9.42	OOR<	OOR<

Supplementary Fig. 4: Soluble MIC induces enhanced expression of tumor- promoting inflammatory cytokines and chemokines in NK cells

(a) Splenic NK cells isolated from *Rag1-/-* mice (expanded in presence of IL-2 for 5 days) were co-cultured with tumor cell lines TC2, TC2-sMICB and TC2-mMICB. Supernatant was collected from 6 hour and 36 hours co-culture timepoints and analyzed by mouse cytokine array assay. Bar graphs showing differential expression of CCL3, CCL4 and IL-10 in mouse NK cells when stimulated with sMIC vs mMIC for shorter versus longer time period.**(b)** Analysis of expression of the mouse NKG2D ligands Rae-1 (Biolegend, 130107), MULT1 (R&D systems, FAB2588A), H60 (R&D systems, FAB1155P) on TC2, TC2-sMICB and TC2-mMICB cells by flow cytometry. **(c)** Evaluation of cytokines and chemokines in the mouse tumor cells cultured alone (without NK cells) in the same experimental settings of main Figure 3. Supernatant was collected after 24hrs of seeding the cells for quantitative cytokine array assay (Eve Technologies).OOR< represents out of range values falling below the standard range of detection. **(d, e)** Splenic NK cells isolated from *Rag1-/-* mice (expanded in presence of IL-2 for 5 days) were co-cultured with tumor cell lines TC2, TC2-sMICB and TC2-mMICB for 18 hours. Gene expression of inflammatory cytokines **(d)**IL-10 and **(e)** GMCSF was analyzed by qPCR. Gene expression was normalized to NK1.1. **(f, g)** Mouse NK cells were stimulated with recombinant sMICB (Sino Biologicals, 10759-H08H) in presence and absence of B10G5 for 18 hours and gene expression of inflammatory cytokines **(f)**IL-10 and **(g)** GMCSF was analyzed by qRT-PCR. Data shown are representative of 3 independent experiments. * represents $p< 0.05$, *** represents $p< 0.001$(Student's t-test; two tailed)

Supplementary Fig. 5

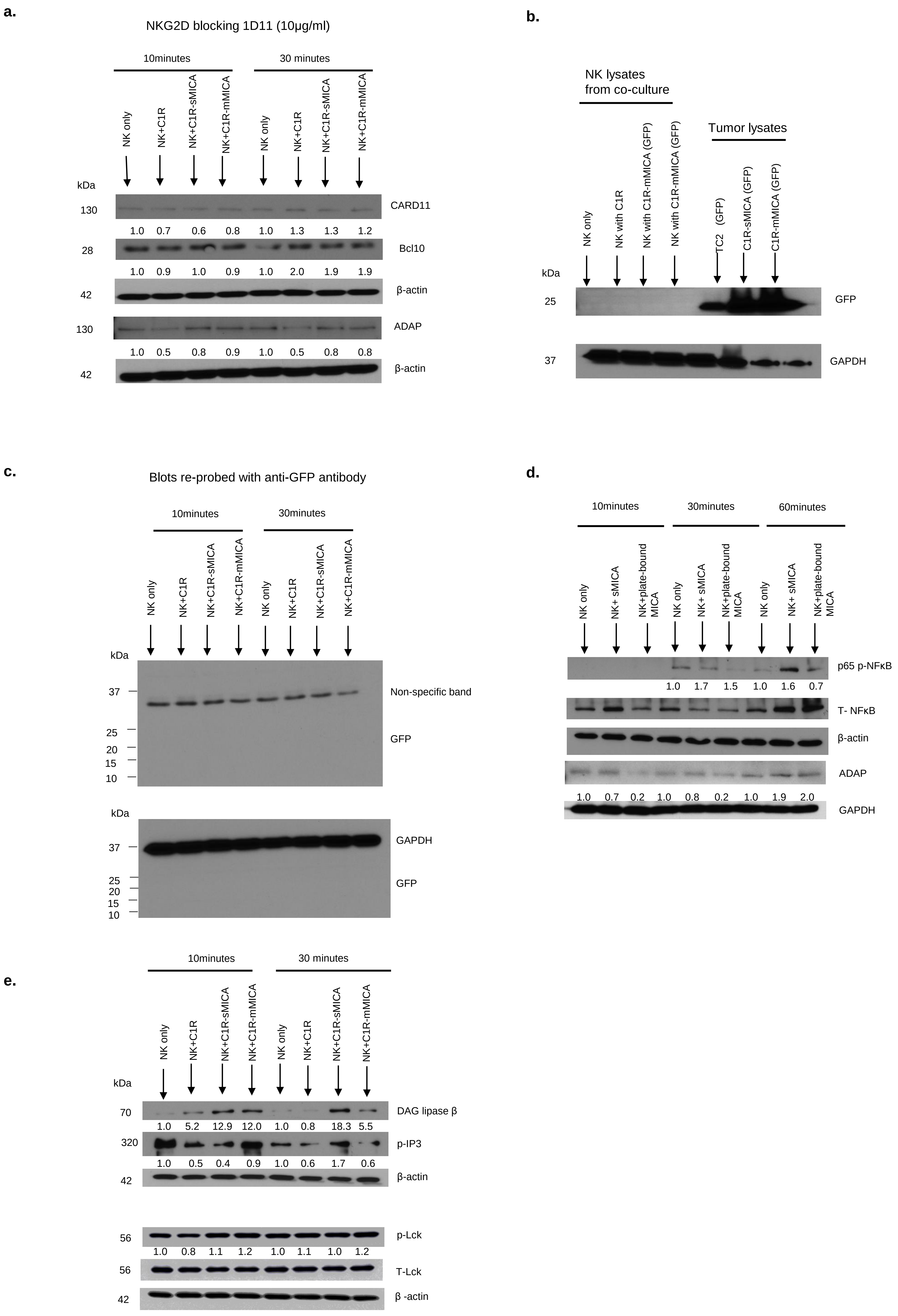

Supplementary Fig. 5: (a) Western blot analysis demonstrating that the differences in the activation of key signaling molecules of cytokine pathways in NK cells upon sMIC and mMIC stimulation were abrogated when the NK cells were pre-incubated with NKG2D blocking antibody 1D11 (10 μg/ml). **(b&c)** Western blot analysis using anti-GFP antibody to demonstrate that there is no contamination from GFP+ tumor cells in the NK cell fraction obtained from the co-culture for lysate preparation. **(d)**Western blot analysis demonstrating that ADAP activation and phosphorylation of the NF-κB subunit p65 were evidently elevated in NK cells stimulated with recombinant sMIC as compared to stimulation with plate-bound MIC (1ug/ml). **(e)** Western blot analysis of DAG lipase β , p-IP3 and Src kinase Lck in human NK cells stimulated with sMIC versus mMIC for 10 minutes and 30 minutes. The numbers represents quantified values after normalization to total proteins or β-actin/GAPDH (and represented as relative to NK only condition). Quantification was performed using Image J software.

Supplementary Fig. 6

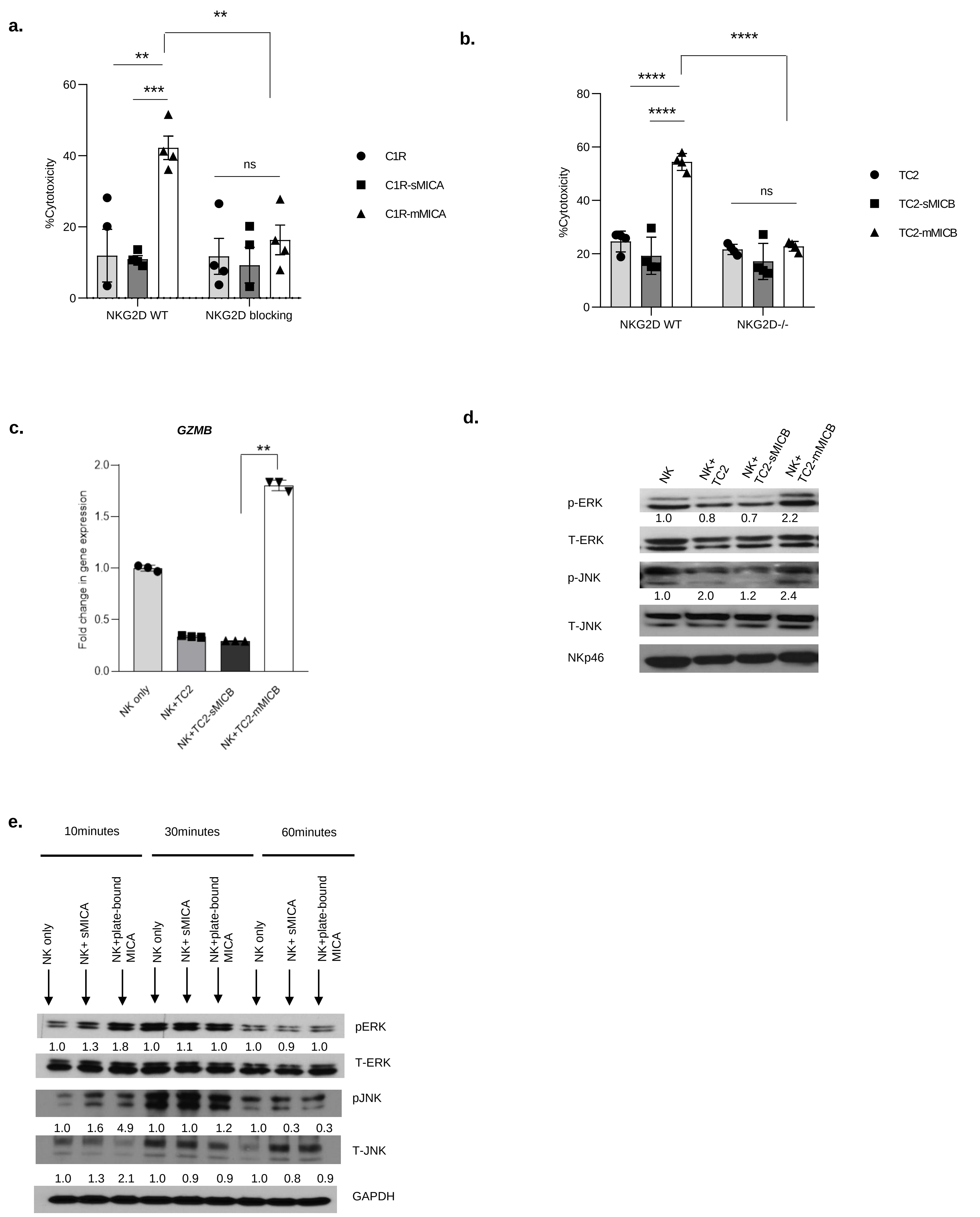

Supplementary Fig. 6: Membrane bound, but not soluble MIC preferentially enhances NK cell cytotoxicity and associated signaling pathways

(a) Cytotoxicity of human NK cells against target cells C1R, C1R-sMICA, C1R-mMICA at E/T ratio of 5:1 for 4 hours, with and without NKG2D blocking (1D11) conditions, assessed by LDH assay. **(b)** Cytotoxicity of mouse NK cells against target cells TC2, TC2-sMICB, TC2-mMICB at E/T ratio of 5:1 for 4 hours, assessed by LDH assay. Splenic NK cells isolated from *Rag1-/-* mice (NKG2D WT) and NKG2D knockout mice (NKG2D-/-) ,activated in presence of IL-2 for 5 days, were co-cultured with tumor cell lines TC2, TC2-sMICB and TC2-MICB.A2 . **(c)** Gene expression analysis of Granzyme B in mouse NK cells analyzed by qPCR. Gene expression was normalized to NK1.1. **(d)** Western blot analysis to evaluate the activation of ERK phosphorylated at Tyr 202/Tyr204 (p-ERK) and JNK phosphorylated at Thr 183/Tyr185 (p-JNK). **(e)** Western blot analysis of ERK and JNK in human NK cells stimulated with recombinant sMIC versus plate-bound MIC (1ug/ml) for different stimulation time points.** p< 0.01, *** p<0.001, **** p< 0.0001, ns: not significant (Student's t-test; two tailed)

Supplementary Fig. 7

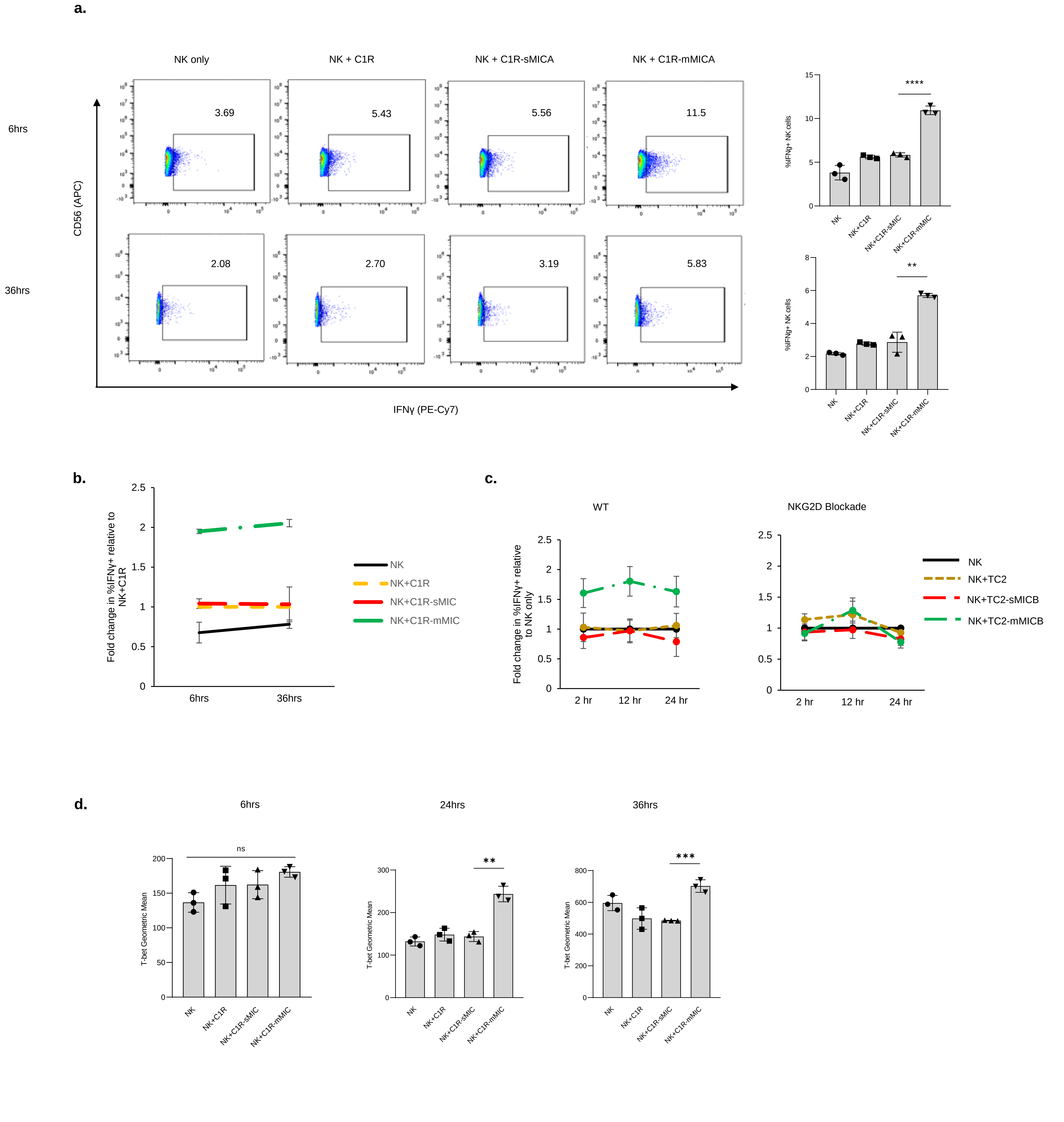

Supplementary Fig. 7: Membrane bound MIC induces higher IFNγ production in NK cells

(a) Representative flow cytometry dot plots and summary bar graphs of the evaluation of IFNγ production by human NK cells co-cultured with C1R, C1R-sMICA, C1R-mMICA cells at E/T ratio of 1:1 for different time points of 6 and 36 hours. IFNγ production was analyzed by intracellular staining using cytofix/cytoperm kit (BD biosciences, 554714) . **(b)** Line graph presentation of the data from **(a)** to highlight the dynamics of stimulation time-dependent dynamics of IFNγ production in human NK cells. Data is presented as fold change in %IFNγ+ NK cells in respective conditions relative to NK+C1R condition .**(c)** Evaluation of IFNγ production by mouse NK cells co-cultured with TC2, TC2-sMICB, TC2-mMICB cells at E/T ratio of 1:1 for different time points of 2, 12 and 24 hours, with (right panel) and without (left panel) NKG2D blocking , analyzed by flow cytometry. Data is presented as fold change in %IFNγ+ NK cells in respective conditions relative to NK only condition. **(d)** Bar graphs demonstrating T-bet expression in human NK cells co-cultured with C1R, C1R-sMICA, C1R-mMICA cells at E/T ratio of 1:1 for different time points of 6, 24 and 36 hours. Staining for T-bet was performed using True Nuclear transcription factor buffer set (Biolegend, 424401). ** represents p< 0.01 (Student’s t-test; two tailed)

Supplementary Fig. 8

a.

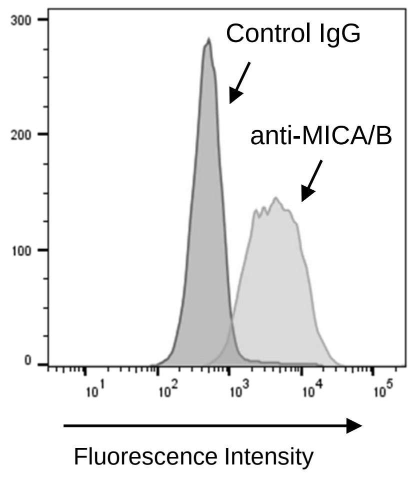

b.

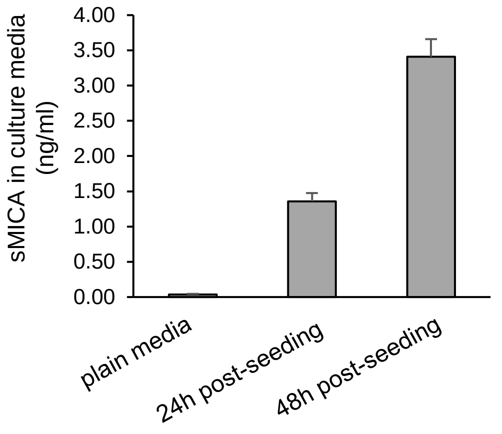

Supplementary Fig. 8: PL12 cell express membrane-bound MIC and shed sMIC. (a) Representative flow cytometry histogram demonstrating that PL12 cells express surface MIC(A/B). Cells were stained with the anti-MIC mAb B10G5. (b) sMICA level in PL12 cell culture as measured with R&D sMICA duo ELISA kits. PL12 cells were seeded in 24 well plate in 2 ml complete media. 100ul of culture media at indicated time were used for ELISA assay.

Supplementary Fig. 9

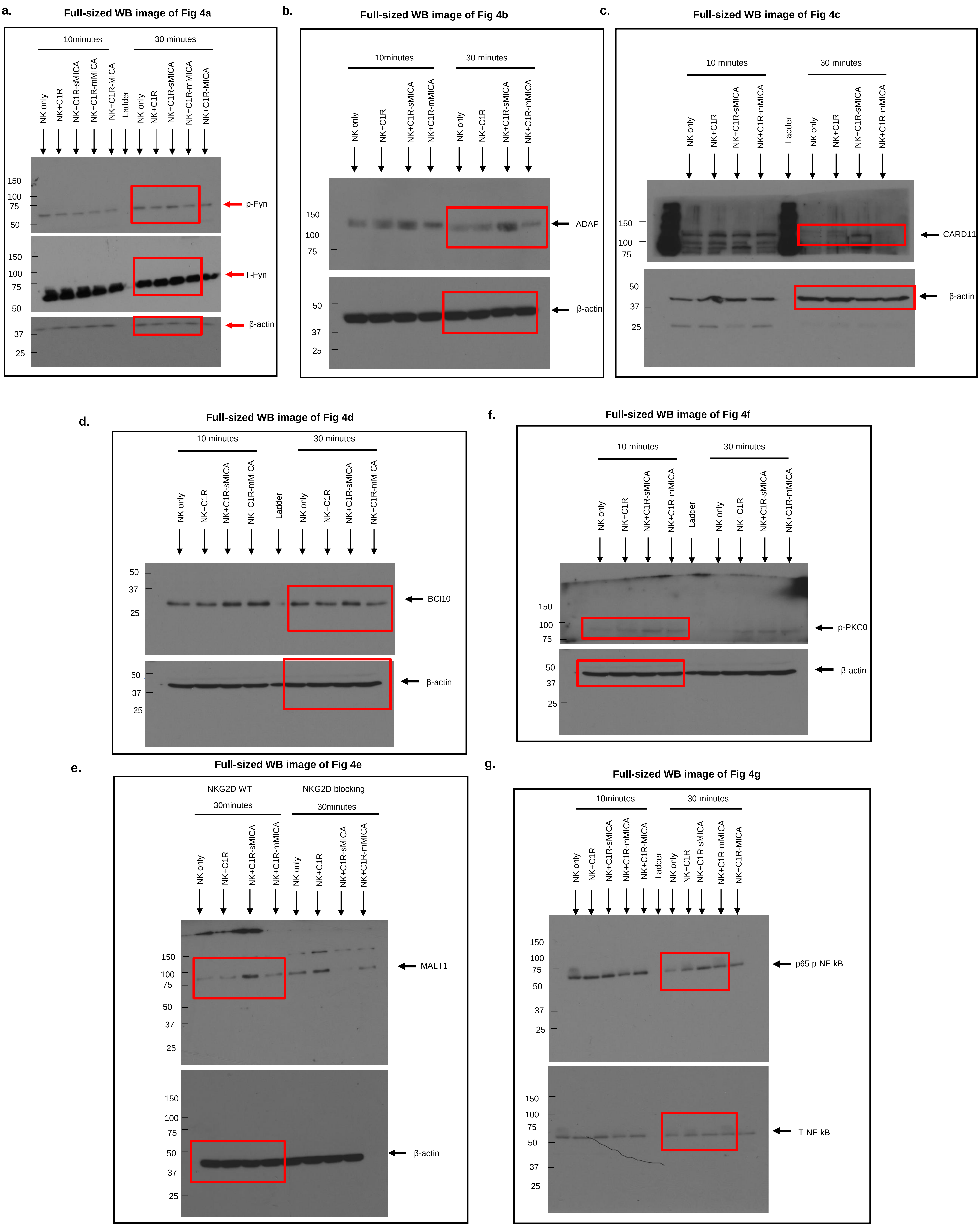

Supplementary Fig. 9: Raw images of Western blots
(a-g) Full-sized raw images of western blots from Figures 4a-4g. Red boxes highlight the areas represented in the figures 4a-4g

Supplementary Fig. 10

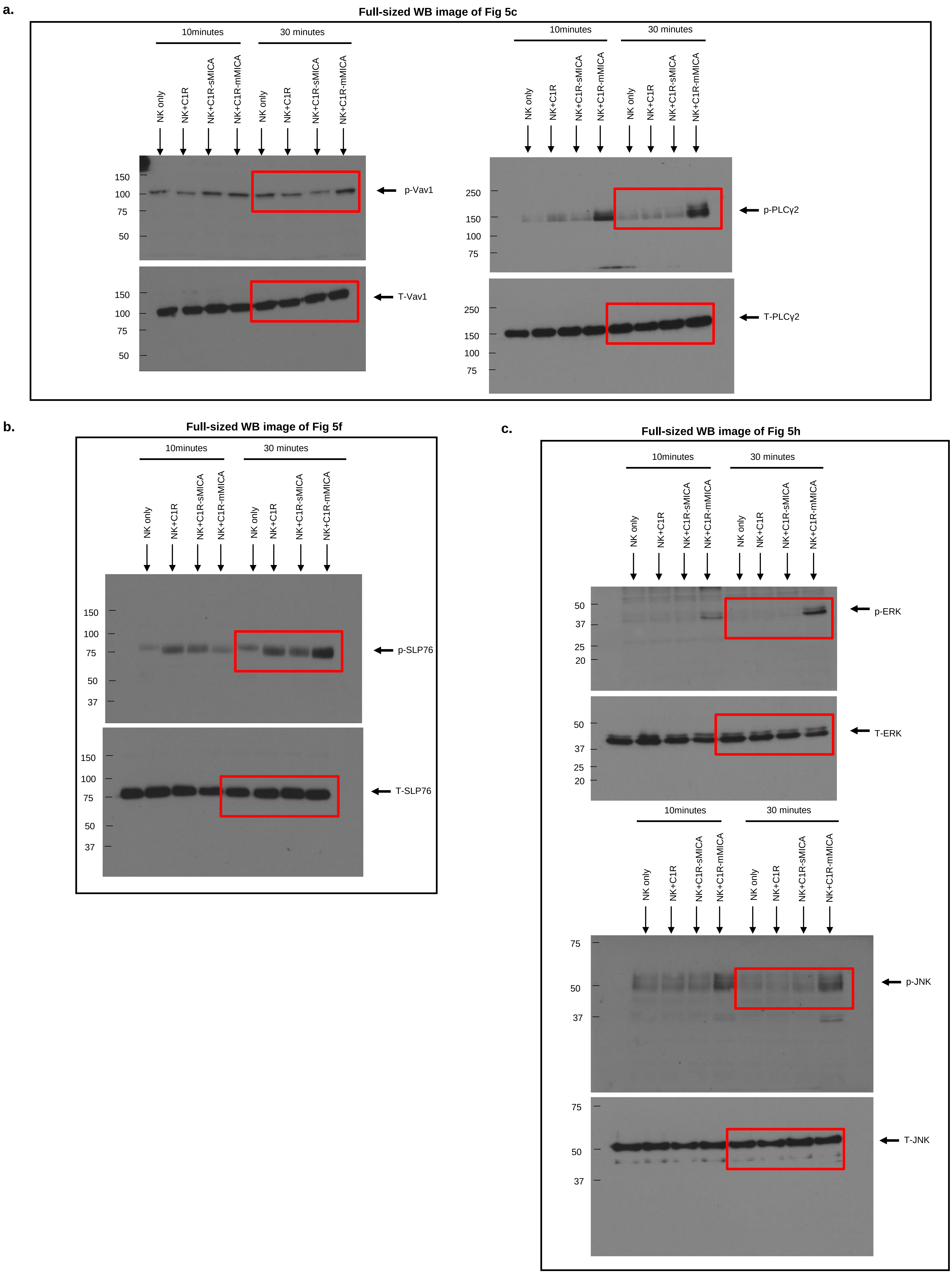

Supplementary Fig. 10: Raw images of Western blots

(a-c) Full-sized raw images of western blots from Figures 5c, 5f and 5h. Red boxes highlight the areas represented in the indicated figures

Supplementary Table 1

Gene	NK cell type	Gene Description	Associated function	Gene Expression sMIC vs Control	Gene Expression anti-sMIC vs sMIC
NCR1	Human	Natural cytotoxicity triggering receptor 1	Cytotoxicity	Downregulated	Upregulated
CD226	Human, Mouse	DNAX Accessory Molecule-1	Cytotoxicity	Downregulated	Upregulated
CD160	Human, Mouse	CD160 antigen	Cytotoxicity	Downregulated	Upregulated
GZMM	Human	Granzyme M	Cytotoxicity	Downregulated	Upregulated
KLRK1	Human, Mouse	NKG2D	Cytotoxicity	Downregulated	Upregulated
IFNg	Mouse	Interferon gamma	Cytotoxicity	Downregulated	Upregulated
Gzmb	Mouse	Granzyme B	Cytotoxicity	Downregulated	Upregulated
IL-10	Human, Mouse	Interleukin-10	Pro-inflammatory	Upregulated	Downregulated
RELB	Human, Mouse	RELB Proto-Oncogene, NF-KB Subunit	Pro-inflammatory	Upregulated	Downregulated
CCL1	Human	Chemokine Ligand 1	Pro-inflammatory	Upregulated	Downregulated
CCL3	Human	Macrophage inflammatory protein-1alpha	Pro-inflammatory	Upregulated	Downregulated
NFKB2	Human, Mouse	Nuclear factor NF-kappa-B p100 subunit	Pro-inflammatory	Upregulated	Downregulated
CCL4	Human, Mouse	Macrophage inflammatory protein-1beta	Pro-inflammatory	Upregulated	Downregulated
Tgfb1	Mouse	Transforming growth factor beta1	Pro-inflammatory	Upregulated	Downregulated
Rela	Mouse	Nuclear factor NF-kappa-B p65 subunit	Pro-inflammatory	Upregulated	Downregulated

Supplementary Table 1: Key differentially expressed cytotoxicity and pro-inflammatory function associated genes identified by bulk RNA sequencing analysis in human and mouse NK cells

Supplementary Table 2

Species	Gene	Primer Sequence
Human	REL B	Fwd: 5'- TGTGGTGAGGATCTGCTTCCAG-3'<br>Rev:5'- TCGGCAAATCCGCAGCTCTGAT-3'
Human	CCL1	Fwd: 5'- ACCAGCTCCATCTGCTCCAATG-3'<br>Rev:5'- TGTGCCTCTGAACCCATCCAAC-3'
Human	TNF	Fwd: 5'- CTCTTCTGCCTGCTGCACTTTG-3'<br>Rev:5'- ATGGGCTACAGGCTTGTCACTC-3'
Human	NFκB2	Fwd: 5'- GGCAGACCAGTGTCATTGAGCA-3'<br>Rev:5'- CAGCAGAAAGCTCACCACACTC-3'
Human	NCR1	Fwd: 5'- CAGCAACTTGCTGGATCTGGTG-3'<br>Rev:5'- AGACGGCAGTAGAAGGTCACCT-3'
Human	CD160	Fwd: 5'- GACCTACCAGTGTTGTGCCAGA-3'<br>Rev:5'- ATCCCGTCACTGTGTAGTTCCC-3'
Human	GZMM	Fwd: 5'- CTCACTGCAGAGAAATGGCTCC-3'<br>Rev:5'- CCTTGATGTGGAAGGTGAGACC-3'
Human	IL-10	Fwd: 5'-GGTTGCCAAGCCTTGTCTGA -3'<br>Rev:5'-AGGGAGTTCACATGCGCCT -3'
Human	CCL4	Fwd: 5'- GCTTCCTCGCAACTTTGTGGTAG-3'<br>Rev:5'- GGTCATACACGTACTCCTGGAC -3'
Human	CCL3	Fwd: 5'- ACTTTGAGACGAGCAGCCAGTG-3'<br>Rev:5'-TTTCTGGACCCACTCCTCACTG -3'
Human	KLRK1	Fwd: 5'- GGTATGAGAGCCAGGCTTCTTG-3'<br>Rev:5'- GAATGGAGCCATCTTCCCACTG-3'
Human	CD226	Fwd: 5'- GGTGATACAGGTGGTTCAGTCAG-3'<br>Rev:5'-GGCTGGATCTTTTCCCACCTCA -3'
Human	Beta-Actin	Fwd: 5'- CACCATTGGCAATGAGCGGTTC-3'<br>Rev:5'- AGGTCTTTGCGGATGTCCACGT -3'
Mouse	GzmB	Fwd: 5'- CCT CCT GCT ACT GCT GAC CT-3'<br>Rev:5'-CAT GTA GGG TCG AGA GTG GG -3'
Mouse	NFκB2	Fwd: 5'- TGCTGATGGCACAGGACGAGAA-3'<br>Rev:5'- GTTGATGACGCCGAGGTACTGA-3'
Mouse	RELB	Fwd: 5'- GTTCTTGGACCACTTCCTGCCT-3'<br>Rev:5'- TAGGCAAAGCCATCGTCCAGGA-3'
Mouse	RELA	Fwd: 5'- TCCTGTTCGAGTCTCCATGCAG-3'<br>Rev:5'- GGTCTCATAGGTCCTTTTGCGC-3'
Mouse	IL-10	Fwd: 5'-ATCGATTTCTCCCCTGTGAA -3'<br>Rev:5'-TGTCAAATTCATTCATGGCCT -3'
Mouse	CD160	Fwd: 5'- GCCACTTTCTCTCCGTTCTAGTC -3'<br>Rev:5'- AGGAAGCCTGAACTGAGAGTGC -3'
Mouse	GMCSF	Fwd: 5'-TGCCTGTCACATTGAATGAA -3'<br>Rev:5'-CCGTAGACCCTGCTCGAATA -3'
Mouse	TNF	Fwd: 5'-CCACCACGCTCTTCTGTCTAC -3'<br>Rev:5'-AGGGTCTGGGCCATAGAACT -3'
Mouse	Tgfb1	Fwd: 5'-GGAGAGCCCTGGATACCAAC -3'<br>Rev:5'-CAACCCAGGTCCTTCCTAAA -3'
Mouse	NK1.1	Fwd: 5'- AAGGTTCACATTGCCAGACA-3'<br>Rev:5'- CACAGCTGCCATTTTCAGTG-3'
Mouse	CCL4	Fwd: 5'- CATGAAGCTCTGCGTGTCTG-3'<br>Rev:5'- GAAACAGCAGGAAGTGGGAG-3'
Mouse	CCL5	Fwd: 5'- CCTGCTGCTTTGCCTACCTCTC-3'<br>Rev:5'- ACACACTTGGCGGTTCCTTCGA-3'
Mouse	KLRK1	Fwd: 5'-CCTATCACTGGATGGGACTGGT -3'<br>Rev:5'- GCTTGAGCCATAGACAGCACAG-3'
Mouse	CD226	Fwd: 5'- CAGACATTGGCATCTACTCCTGC-3'<br>Rev:5'-GACATCTTGTCCAGGTTCTGCAG -3'
Mouse	Beta-actin	Fwd: 5'- CATTGCTGACAGGATGCAGAAGG -3'<br>Rev:5'- TGCTGGAAGGTGGACAGTGAGG-3'
