# Supplementary material for: Tumor-derived NKG2D ligand sMIC reprograms NK cells to an inflammatory phenotype through CBM signalosome activation
Source: Commun Biol. 2021 Jul 22;4:905. doi: 10.1038/s42003-021-02440-3 (PMC8298432; doi:10.1038/s42003-021-02440-3)
Supplement: Supplementary file 3 — Description of Supplementary Files [file 42003_2021_2440_MOESM3_ESM.pdf]

Description of Additional Supplementary Files

File name: Supplementary Data 1

Description: An excel file containing the following source data: Dataset Fig1a-1d, Dataset Fig 1e, Dataset Fig 2, Dataset Fig 3, Dataset Fig 4, Dataset Fig 5, Dataset Fig 6, Dataset Fig 7.
