# Supplementary material for: Tumor-derived NKG2D ligand sMIC reprograms NK cells to an inflammatory phenotype through CBM signalosome activation
Source: Commun Biol. 2021 Jul 22;4:905. doi: 10.1038/s42003-021-02440-3 (PMC8298432; doi:10.1038/s42003-021-02440-3)
Supplement: Supplementary file 5 — Reporting Summary [file 42003_2021_2440_MOESM5_ESM.pdf]

nature research

Corresponding author(s): Jennifer Wu

Last updated by author(s): June 24, 2021

Reporting Summary

Nature Research wishes to improve the reproducibility of the work that we publish. This form provides structure for consistency and transparency in reporting. For further information on Nature Research policies, see our Editorial Policies and the Editorial Policy Checklist.

Statistics

For all statistical analyses, confirm that the following items are present in the figure legend, table legend, main text, or Methods section.

n/a	Confirmed	
[ ]	[x]	The exact sample size (*n*) for each experimental group/condition, given as a discrete number and unit of measurement
[ ]	[x]	A statement on whether measurements were taken from distinct samples or whether the same sample was measured repeatedly
[ ]	[x]	The statistical test(s) used AND whether they are one- or two-sided<br>*Only common tests should be described solely by name; describe more complex techniques in the Methods section.*
[ ]	[x]	A description of all covariates tested
[x]	[ ]	A description of any assumptions or corrections, such as tests of normality and adjustment for multiple comparisons
[ ]	[x]	A full description of the statistical parameters including central tendency (e.g. means) or other basic estimates (e.g. regression coefficient) AND variation (e.g. standard deviation) or associated estimates of uncertainty (e.g. confidence intervals)
[ ]	[x]	For null hypothesis testing, the test statistic (e.g. *F*, *t*, *r*) with confidence intervals, effect sizes, degrees of freedom and *P* value noted<br>*Give P values as exact values whenever suitable.*
[x]	[ ]	For Bayesian analysis, information on the choice of priors and Markov chain Monte Carlo settings
[x]	[ ]	For hierarchical and complex designs, identification of the appropriate level for tests and full reporting of outcomes
[x]	[ ]	Estimates of effect sizes (e.g. Cohen's *d*, Pearson's *r*), indicating how they were calculated

Our web collection on statistics for biologists contains articles on many of the points above.

Software and code

Policy information about availability of computer code

Data collection	The numeric data were collected using Excel and image data by ImageJ software
Data analysis	Packages available in "R" were used to create the heatmaps, UMAPs and clustering for bulk RNA sequencing and single cell RNA sequencing data. Flow cytometry data was analyzed using FlowJo sofware.

For manuscripts utilizing custom algorithms or software that are central to the research but not yet described in published literature, software must be made available to editors and reviewers. We strongly encourage code deposition in a community repository (e.g. GitHub). See the Nature Research guidelines for submitting code & software for further information.

Data

Policy information about availability of data

All manuscripts must include a data availability statement. This statement should provide the following information, where applicable:

- Accession codes, unique identifiers, or web links for publicly available datasets
- A list of figures that have associated raw data
- A description of any restrictions on data availability

Data Availability Statement is included in the manuscript.

Field-specific reporting

Life sciences study design

All studies must disclose on these points even when the disclosure is negative.

Sample size	Sample sizes were chosen according to the standard practice (at least 3 replicates in each sample condition)
Data exclusions	No data was excluded from consideration
Replication	For quantitative assays and western blots, biological and technical repeats for at least n=3 were applied (unless noted otherwise). Reported results were consistently replicated across multiple experiments and the findings were consistent across different types of experiments.<br><br>Considering the potential variability in the sources of reagents like recombinant proteins, different sources of recombinant soluble MIC protein were tested for consistency.
Randomization	Both male and female Rag1-/- mice were used for the isolation of splenic NK cells<br><br>For the in-vivo study, animals were randomized into four therapeutic groups with equal numbers of male (n=3) and female (n=3) mice in each group, after tumors reached 50-75 mm3 in size.<br><br>Commercially available frozen PBMCs of random donors were used for NK cell expansions
Blinding	When performing multiplex cytokine array experiments, polyfunctionality assay or ELISA, samples were blinded and coded to prevent bias.

Reporting for specific materials, systems and methods

We require information from authors about some types of materials, experimental systems and methods used in many studies. Here, indicate whether each material, system or method listed is relevant to your study. If you are not sure if a list item applies to your research, read the appropriate section before selecting a response.

Materials & experimental systems

n/a	Involved in the study	
☐	☒	Antibodies
☐	☒	Eukaryotic cell lines
☒	☐	Palaeontology and archaeology
☐	☒	Animals and other organisms
☒	☐	Human research participants
☒	☐	Clinical data
☒	☐	Dual use research of concern

Methods

n/a	Involved in the study	
☒	☐	ChIP-seq
☐	☒	Flow cytometry
☒	☐	MRI-based neuroimaging

Antibodies

Antibodies used	All antibodies used for flow cytometry assay and Western Blots are listed in the materials and methods section and supplementary section with the supplier name and catalog number. The B10G5 antibody was produced through BioXcell Inc. from the hybridoma stock.
Validation	Most of the antibodies were used at the same concentration as per manufacturers recommendation and titrated as necessary. The B10G5 antibody was validated by SPR binding assay to confirm affinity for sMIC.

Eukaryotic cell lines

Policy information about cell lines

Cell line source(s)	Parental TRAMP-C2 (TC2) cell line was purchased from ATCC.<br>Parental C1R cells were purchased from ATCC<br>PL 12 cells provided by Dr. Nicholas Cacalano, UCLA Jonsson Comprehensive Cancer Center
Authentication	Cell lines were routinely validated for stable transduction using flow cytometry
Mycoplasma contamination	All cell lines used in the current study were routinely tested for mycoplasma contamination using PlasmoTest Mycoplasma Detection Kit (InvivoGen, rep-pt1)
Commonly misidentified lines (See ICLAC register)	*Name any commonly misidentified cell lines used in the study and provide a rationale for their use.*

Animals and other organisms

Policy information about studies involving animals; ARRIVE guidelines recommended for reporting animal research

Laboratory animals	8-10 weeks old NSG mice<br>Rag1-/- mice of 8-12 weeks<br>TRAMP/MICB mice (20-32weeks old)
Wild animals	Study did not involve wild animals
Field-collected samples	Study did not involve samples collected in the field
Ethics oversight	All animal studies were approved by the Institutional Animal Care and Use Committee (IACUC) committee of Northwestern University.

Note that full information on the approval of the study protocol must also be provided in the manuscript.

Flow Cytometry

Plots

Confirm that:

- [x] The axis labels state the marker and fluorochrome used (e.g. CD4-FITC).
- [x] The axis scales are clearly visible. Include numbers along axes only for bottom left plot of group (a 'group' is an analysis of identical markers).
- [ ] All plots are contour plots with outliers or pseudocolor plots.
- [x] A numerical value for number of cells or percentage (with statistics) is provided.

Methodology

Sample preparation	Sample preparation is described in details in the Methods section
Instrument	BD Fortessa cytometer and BD FACS Aria III
Software	FACSDiva for data collection FlowJo software for data analysis
Cell population abundance	For single cell and bulk RNAseq experiments, cell viability of >90% was applied as the mandatory threshold.
Gating strategy	Example of gating strategy for Fig 2h is shown in Supplementary Figure S3

- [x] Tick this box to confirm that a figure exemplifying the gating strategy is provided in the Supplementary Information.
